# Genome sequence of Leptolyngbya phage Dor1, a cyanophage induced from a fish pond

**DOI:** 10.1128/mra.01098-24

**Published:** 2024-12-20

**Authors:** Esther Cattan-Tsaushu, Lilach Pessen, Sarit Avrani

**Affiliations:** 1 Department of Evolutionary and Environmental Biology and The Institute of Evolution, University of Haifa, Haifa, Israel; DOE Joint Genome Institute, Berkeley, California, USA

**Keywords:** cyanobacteria, cyanophages, lysogeny, prophage

## Abstract

Leptolyngbya phage Dor1 was induced by mitomycin C from a fishpond and was isolated on *Leptolyngbya boryana* IU 594. The 41,522-bp genome of Leptolyngbya phage Dor1 has 93.77% intergenomic similarity with Leptolyngbya phage LPP-1; however, unlike LPP-1, Dor1 carries an HNH endonuclease in its DNA polymerase gene.

## ANNOUNCEMENT

Cyanophages of the family *Saffermanviridae* are non-marine, short-tailed phages, most of which can lysogenize their hosts despite lacking an identifiable integrase gene (1). The first described cyanophage, Leptolyngbya phage LPP-1 (hereafter LPP-1), is a member of this family (2). To identify additional temperate cyanophages, a water sample was collected in November 2023 from Dor Aquaculture Research Station, Israel (32°36'31.7" N 34°55'45.1"E). The sample was filtered through a 0.22-µm filter, and the retained material was incubated overnight in BG-11 medium containing mitomycin C (1 g/L) (3). The resulting lysate was filtered (0.45 µm), washed three times with BG-11 to remove residual mitomycin C, and concentrated 20-fold using a 100-kDa centrifugal filter (BIOFIL). The concentrated lysate was used to inoculate a mid-log culture of *Leptolyngbya boryana* IU 594, with the flow-through and a non-induced sample serving as negative controls. Lysis occurred only in the culture inoculated with the induced sample, indicating phages were induced from the original sample. Dilutions of the lysate were plated with the host in BG-11 medium with low-melting-point agarose. A single plaque was transferred into a liquid culture of the host. The isolated phage was named Leptolyngbya phage Dor1 (hereafter Dor1).

DNA was extracted from the lysate of the isolated phage using the PRESTO Bacteria Genomic DNA kit. A DNA library was prepared from 1 ng of extracted DNA using the TruePrep DNA Library Prep Kit V2 (VAZYME) following the procedure in (4), with 13 amplification cycles. The whole genome was sequenced on a NovaSeq 6000 system (Illumina, 2 × 150 bp), generating approximately 500,000 reads. Sequence quality was assessed with FastQC (5), followed by adapter trimming using Trim Galore (6), and trimmed reads were reassessed with FastQC. Assembly was performed using SPAdes with the parameters: phred offset 33, 24 threads, only-assembler mode, and k-mer sizes of 127, 77, 55, and 105 (6). Open reading frames (ORFs) were predicted using Prodigal (7), annotation was performed by integrating function predictions from Prodigal, and blastp searches were performed against the protein n/r database (8) and HHpred (referencing the UniProt-SwissProt-viral70_3_Nov_2021 database) (9). All software tools were run with default parameters, unless otherwise stated.

The assembled genome of Dor1 is 41,522 bp in length, with an average coverage of 1,435-fold and a GC content of 52.2%. Of the 49 predicted genes, 59% (29) encode proteins of unknown function, 16% (8) are structural, and 25% (10) have other functional annotations. Comparative genomic analysis using VIRIDIC (11) revealed that LPP-1 is the closest relative of Dor1, with an intergenomic similarity of 93.77% based on blastn alignment. Therefore, according to the criteria in (12), they represent distinct species within the genus *Morrisvirus* of the *Saffermanviridae* family. This similarity to LPP-1 enabled the identification of Dor1’s genome termini. Dor1 carries four unique genes not found in the LPP-1 genome. Three of these encode proteins of unknown function, while the fourth is an HNH endonuclease embedded within the gene encoding the phage’s DNA polymerase. A similar gene arrangement has been observed in another member of this family, Pf-WMP3 (10).

## Data Availability

The Whole Genome Shotgun project has been deposited in GenBank under the accession no. PQ195596.1, BioProject accession no. PRJNA1168992, SRA accession no. SRR30889153, and BioSample accession no. SAMN44063328.
